# On the Efficacy of Blood-Letting in Rigidity of the Os Externum

**Published:** 1807-10

**Authors:** Wm. Dewees


### *On the Efficacy of Blood-letting in Rigidity of the Os Externum. By* Dr. Wm. Dewees.

### Case I.

On the 12th September, 1798, I was requested to visit the wife of Samuel Griffith, in consultation with Dr. Jones. Mrs. G——, I was informed by the Doctor, had been in labour sixteen hours; the waters were evacuated early in her labour; her pains frequent and brisk; but, there was not the least disposition in the soft parts to dilate.

I sat down to examine our patient, and found the os externum scarcely large enough to admit the finger, and it was mounted up closely against the symphysis pubis, in consequence of the perineum being very much distended by the head of the child. The os uteri was rigid and but little opened—a kind of bridle or small column of flesh ran from the inferior edge of the os pubis, and lost itself in the perineum below; against this, the head was firmly pressed. The head was situated naturally, and so far advanced, that the vertex was about to emerge from under the arch of the pubes, covered with the uterus; it had been thus fixed for nearly six hours previous to my seeing this patient; and all that had been done, was, the occasional exhibition of tinct. opii. with steady pressure against the perineum to prevent the escape of the head through it. In this situation of affairs what was to be done?

My ingenious and much lamented friend, Dr. E. Smith of New York, immediately after the receipt of my former case, suggested the trial of an infusion of tobacco in similar cases, to supersede the use of such extensive bleeding as had been employed in it; affirming its effects were very similar to those produced by copious blood-letting—such as nausea, vomiting, syncope, and consequently relaxation. The idea pleased me, and I was determined to employ it the first opportunity: The case under consideration I believed to be as favourable a one as could occur, and accordingly proposed it to Dr. Jones; he cheerfully acquiesced in its trial; a strong infusion was made of the tobacco, and a quantity of it with some difficulty, after several ineffectual trials, was thrown up the rectum. It produced great sickness, vomiting and fainting; but the desired relaxation did not take place; we waited some time longer, but with no better success. In the course of an hour, or an hour and a half, the more distressing symptoms produced produced by the infusion wore off; and resolving to give the remedy every chance in our power, we got our patient with some difficulty, to consent to another application of it. Its effects were as before—great distress—without the smallest benefit; the soft parts remaining equally rigid, as before its exhibition.

Supposing the bridle just spoken of might have some influence on the developement of the external parts, I divided it, but without any evident good resulting from it. I now proposed the remedy that had so completely succeeded in a former case,—bleeding nearly to fainting. This was consented to. We had our patient placed on her feet, taking care to have the perinæum well guarded during the operation. Upon taking away about ten ounces of blood she became very faint;* she was immediately laid upon her bed; the most complete relaxation had taken place; the forceps were applied, and our patient was delivered in a very few minutes of a fine healthy girl. The mother was put comfortably to bed, and every thing went on in the ordinary way until the sixth day, when she was seized with a violent cholera morbus and convulsions, (to which complaints she was subject) and died in twelve hours.

This

* The subject of this case was a delicate woman, and wont to become very faint upon the loss of a little blood. The disposition to syncope has frequently aided me in obstetric cases, where this state was an object in practice; it is well to inquire if this peculiarity exists, as, taking advantage of it, by placing our patients in an erect posture, we are enabled to effect by the loss of a few ounces of blood, what could not be obtained in a supine one by the loss of very many. This circumstance may also aid the timid practitioner, who may be afraid to venture on the great depletion that sometimes becomes necessary to produce this effect, when this situation is not attended to. I have therefore always placed my patients on their feet, when practicable, before I opened the vein. We may also spare ourselves a great deal of trouble, and our patients much anxiety, in cases of tedious labour, from the unwillingness of the soft parts to dilate, where this disposition to syncope from the loss of a few ounces of blood when in an erect posture obtains, by taking advantage of this peculiarity. Few women object to being bled during labour, nay, they for the most part think it indispensable when the labour has been protracted. The consent, therefore, of the patient and her friends is easily obtained; all we have to do is, to conceal from them our wish to have her become faint. Should she faint we can readily satisfy them, by calling to their recollection, it is common to her from bleeding, and that it will be useful to her. In this way, we can very often shorten a labour many hours, when we might not be able to obtain the consent of our patient, or we ourselves think it necessary, to bleed her to the extent of forty or fifty ounces.

This Case, notwithstanding its ultimately unfortunate termination, fully establishes the influence of blood-letting in this very distressing kind of rigidity; and proves it to act differently from tobacco, notwithstanding the latter produces nausea, vomiting, and syncope; and also, that the quantity of blood lost, in some instances, may be very small, to induce the desired relaxation.

We conceive that no possible blame can be attached to the bleeding in this case, as the woman was very brave until the sixth day, when a disease, to which she was subject, supervened, and carried her off.

### Case II.

On the 26th of September, 1800, I was called to the wife of Mich. Falkrod, at Frankford, in consultation with Dr. Ruan. She had been in labour twelve or fourteen hours, with her second child;* the pains frequent and strong; the waters discharged some time; the head was situated favourably, and completely occupied the vagina, the perineal tumour large; the os externum not larger than a common finger ring; admitting the finger with some difficulty in the absence of pain; during pain thrown up against the inferior edge of the pubes in such a manner, as not to admit the finger, or allow it to be retained if previously introduced. Externally, a strong cicatrix was found running to the very verge of the anus; internally, it could be traced further. This cicatrix prevented the unfolding of the external parts so effectually, that the repeated efforts of the uterus, for several hours, were insufficient to make them yield, though the head had been closely applied to them during that period.

This patient was a strong healthy woman; considerable fever had been excited; the pulse strong, frequent, and hard. I proposed bleeding ad deliquium, to which Dr. Ruan consented. We immediately opened a vein, and took about forty ounces of blood; but as her pains were so rapid, we were obliged to take it from her in a recumbent posture, and no disposition to syncope was manifested. This quantity, however, had some effect, as there was evidently a beginning relaxation, and an abatement of the

* With the first, she had suffered an extensive laceration of the perineum. It is somewhat remarkable, that all the cases I have related, were in the same neighbourhood, and all had been under the care of the same physician.

the violence and frequency of the pains. We now agreed upon a second bleeding, and to have it taken in an erect situation We with some difficulty effected this, when, upon taking five and twenty or thirty ounces more, she fainted; she was laid upon the bed, and in a few minutes by the forceps, was delivered of a fine healthy boy. Our patient recovered rapidly without accident, or draw-back.

### Case III.

February 26, 1803, I was called to the same woman in labour with her third child. The same circumstances attended, and the same remedy was employed, with a similar result. This case was witnessed by Mr. Bond, an ingenious young gentleman of Baltimore.

